# Supplementary material for: Dietary Inulin to Improve SARS-CoV-2 Vaccine Response in Kidney Transplant Recipients: The RIVASTIM-Inulin Randomised Controlled Trial
Source: Vaccines (Basel). 2024 Jun 3;12(6):608. doi: 10.3390/vaccines12060608 (PMC11209582; doi:10.3390/vaccines12060608)
Supplement: Supplementary file 1 [file vaccines-12-00608-s001.zip › vaccines-3017079-supplementary-figures.pdf]

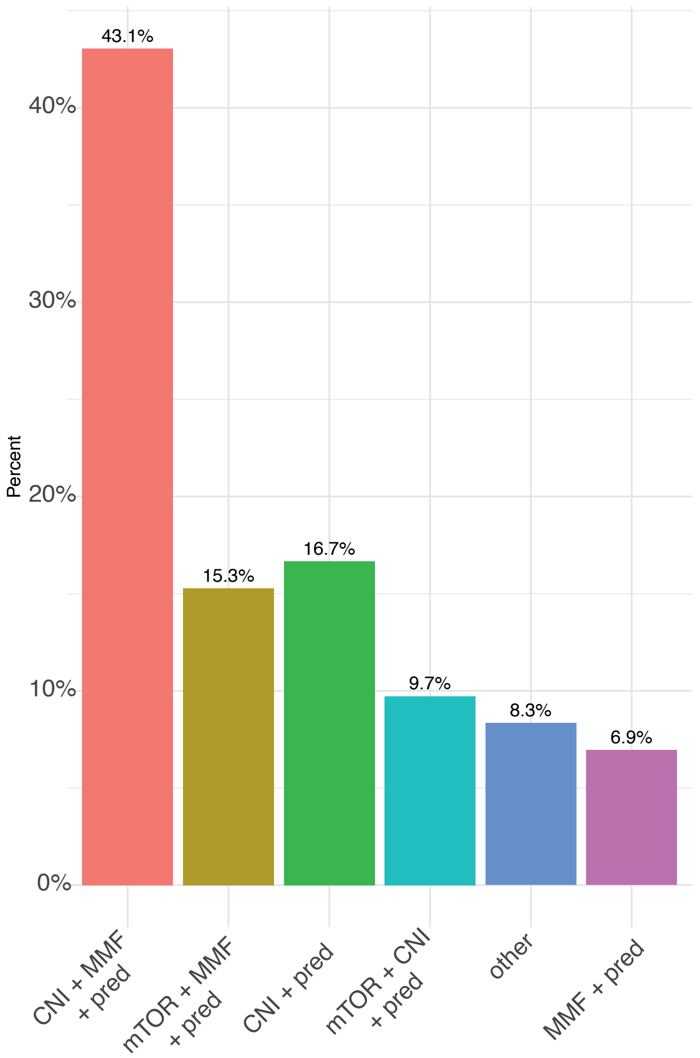

Supplementary Figure S1: Immunosuppression used in kidney transplant recipients

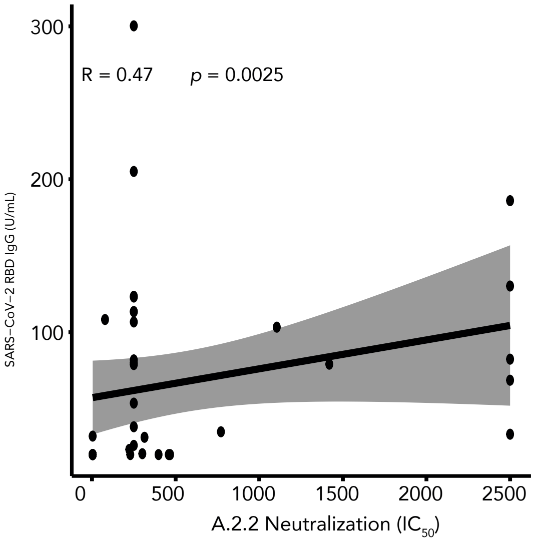

Supplementary Figure S2. Correlation between neutralising antibody by surrogate virus neutralisation test (sVNT) and anti-spike RBD IgG (Spearman's r=0.47, p<0.003

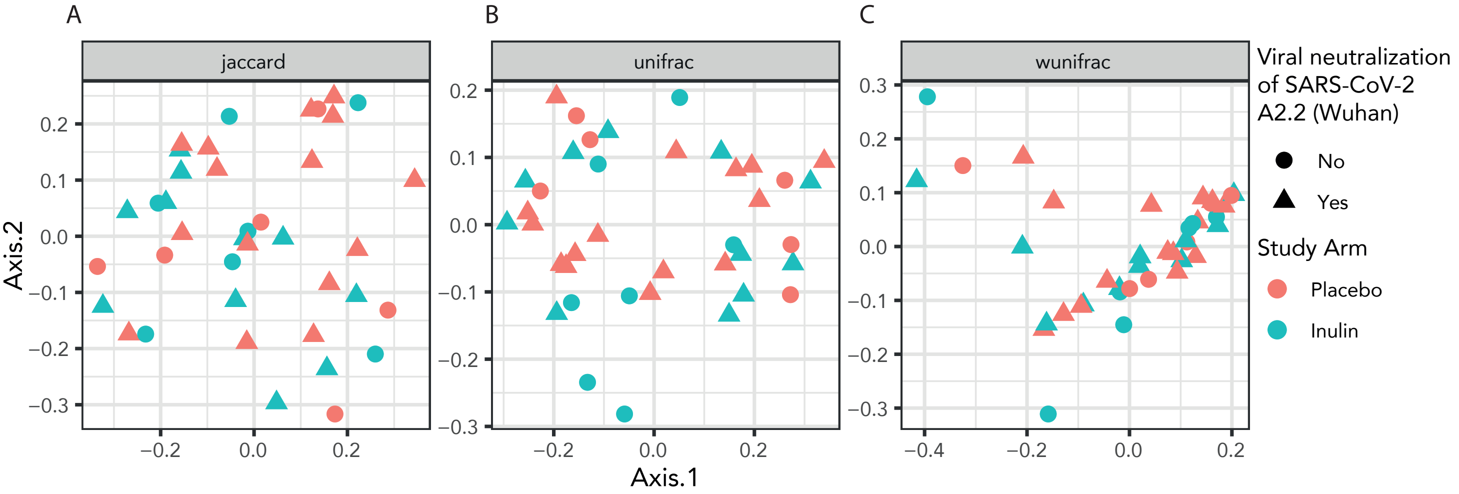

Supplementary figure S3: The community composition at the time of a third COVID-19 vaccine was not associ-ated with the development of seffective SARS-CoV-2 viral neutralisatio, when assessed using both phyloge-netic and non-[hylogenetic measures,

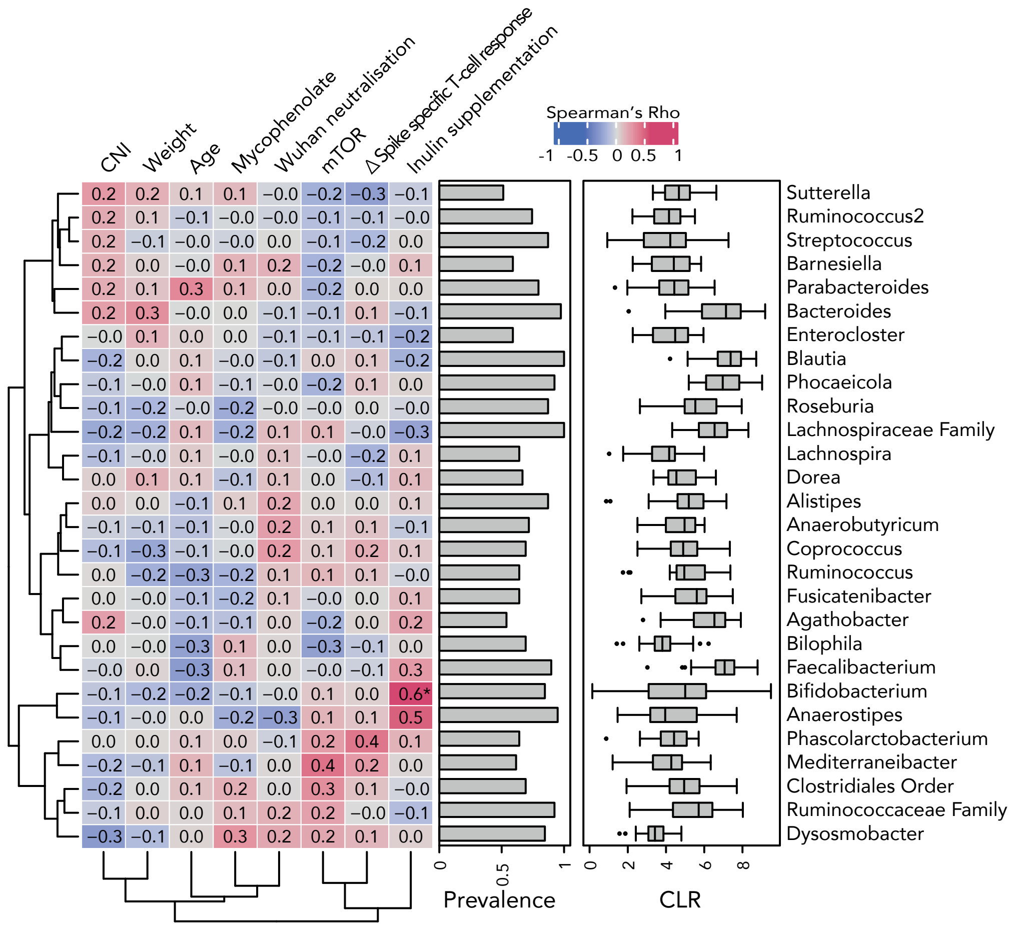

Supplementary figure S4: Spearman's correlation between microbial genus abundance and the immune response to a third SARS-CoV2-vaccination, clinical phenotypes, and immunosupression use. Bifidobacterium*inulin *p*.adj. = 0.028
