# Supplementary material for: Dietary Inulin to Improve SARS-CoV-2 Vaccine Response in Kidney Transplant Recipients: The RIVASTIM-Inulin Randomised Controlled Trial
Source: Vaccines (Basel). 2024 Jun 3;12(6):608. doi: 10.3390/vaccines12060608 (PMC11209582; doi:10.3390/vaccines12060608)
Supplement: Supplementary file 1 [file vaccines-12-00608-s001.zip › vaccines-3017079-supplementary.pdf]

Supplementary Materia

Supplementary Table S1. GSRS scores.

		Inulin	Placebo	p-value
		N=34	N=31	
Baseline	Participant experiencing symptom	n (%)	n (%)	
	Abdominal pain	10 (34)	8 (31)	0.77
	Reflux	7 (24)	7 (27)	0.81
	Diarrhoea	12 (41)	10 (38)	0.83
	Indigestion	17 (59)	13 (50)	0.52
	Constipation	9 (32)	8 (31)	0.91
	Any GIS symptom	20 (69)	19 (73)	0.74
	Mean score	Mean (sd)	Mean (sd)	
	Abdominal pain	1.4 (0.7)	1.2 (0.4)	0.28
	Reflux	1.3 (0.7)	1.3 (0.7)	0.93
	Diarrhoea	1.5 (0.9)	1.4 (0.9)	0.75
	Indigestion	1.5 (0.9)	1.3 (0.5)	0.35
	Constipation	1.4 (0.9)	1.3 (0.5)	0.56
	Global	1.5 (0.7)	1.3 (0.4)	0.42
Week4	Participant experiencing symptom	n (%)	n (%)	
	Abdominal pain	13 (45)	10 (42)	0.82
	Reflux	10 (34)	5 (20)	0.24
	Diarrhoea	17 (59)	13 (52)	0.63
	Indigestion	23 (79)	16 (64)	0.21
	Constipation	7 (24)	11 (44)	0.12
	Any GIS symptom	25 (86)	19 (79)	0.50
	Mean score	Mean (sd)	Mean (sd)	
	Abdominal pain	1.4 (0.7)	1.3 (0.6)	0.63
	Reflux	1.4 (0.8)	1.3 (0.8)	0.55
	Diarrhoea	2.2 (1.5)	1.5 (0.8)	0.028
	Indigestion	2.4 (1.2)	1.6 (0.9)	0.007
	Constipation	1.3 (0.7)	1.5 (1.1)	0.48
	Global	1.9 (0.8)	1.4 (0.5)	0.022
Week 8	Participant experiencing symptom	n (%)	n (%)	
	Abdominal pain	10 (32)	6 (27)	0.70
	Reflux	8 (26)	2 (9)	0.13
	Diarrhoea	18 (58)	10 (45)	0.36
	Indigestion	21 (68)	12 (55)	0.33
	Constipation	10 (32)	10 (45)	0.33
	Any GIS symptom	27 (87)	18 (82)	0.60
	Mean score	Mean (sd)	Mean (sd)	
	Abdominal pain	1.5 (0.7)	1.1 (0.3)	0.063
	Reflux	1.4 (1.0)	1.1 (0.4)	0.15
	Diarrhoea	2.0 (1.5)	1.6 (0.9)	0.27
	Indigestion	2.1 (1.2)	1.4 (0.6)	0.019
	Constipation	1.4 (0.8)	1.3 (0.4)	0.75
	Global	1.8 (0.9)	1.3 (0.4)	0.037

Supplementary Table S2. EQ-5D-5L Index – overall health status.

Visit	Inulin		Placebo		p-value*
	N	Mean (SD)	N	Mean (SD)	
Week 0	30	0.88 (0.17)	26	0.92 (0.15)	0.34
Week 4	29	0.93 (0.12)	25	0.96 (0.06)	0.60
Week 8	31	0.92 (0.13)	22	0.95 (0.08)	0.24

UK value set and scoring algorithm were used to calculate utility scores as an Australian scoring algorithm is not yet available for the 5L. *p-value derived from Mann Whitney U Test.

Supplementary Table S3. Summary of adverse events in the RIVASTIM-inulin trial.

	Inulin	Placebo	P-value
	N=34	N=31	
Patients with any AEs	14 (41)	9 (29)	0.31
Patients with severe AEs	2 (6) (Severe bloating; Faecal incontinence due to diarrhoea)	2 (6) (Elective fistula ligation and skin cancer removal; Fevers of unclear focus)	0.92
Patients with Serious AE requiring expedited reporting	0 (0)	0 (0)	
Patients with AE leading to death	0 (0)	0 (0)	
AEs	**17**	**9**	
Gastrointestinal	13	6	0.15
Surgical procedures	2	1	0.66
Cardiac	1	0	0.36
Infection	1	1	0.90
General/injection administration site	0	1	0.27
AE attribution	**17**	**9**	
Neither the vaccine nor the treatment group	6	1	
Receiving the vaccine (3rd dose)	0	1	
Intervention (Inulin)	3	3	
Control (Placebo)	6	3	
Both the vaccine AND the intervention/control	0	0	
Missing attribution	2	1	

Supplementary Table S4. Summary of adverse events of special interest in the RIVASTIM-inulin trial.

Factor	Arm	N	Mean change (Final visit – baseline)	SD	p-value
Serum creatinine	Inulin	34	0.0	4.9	
	Placebo	29	2.1	3.2	
	Difference between 2 arms		-2.1	6.1	0.73
eGFR	Inulin	34	-3.3	1.9	
	Placebo	29	-0.6	1.5	
	Difference between 2 arms		-2.7	2.4	0.27
Urine ACR	Inulin	30	-6.2	6.0	
	Placebo	26	-2.7	1.6	
	Difference between 2 arms		-3.4	6.6	0.61

Supplementary Table S5. Proportion of participants that reach a threshold of serological anti-SARS-CoV-2 RBD Ig antibody ≥ 100 units/mL.

	Inulin	Placebo	p-value
	n =34	n =31	
Anti-RBD Ig at 8 weeks			0.61
< 0.8 units/mL	3 (9)	1 (3)	
0.8- 100 units/mL	11 (32)	11 (37)	
≥ 100 units/mL	18 (53)	15 (50)	
Missing	2 (6)	4 (13)	
Baseline Anti-RBD Ig < 0.8 units/mL	11	9	
Anti-RBD Ig at 8 weeks			0.28
< 0.8 units/mL	3 (27)	1 (11)	
0.8- 100 units/mL	7 (64)	7 (78)	
≥ 100 units/mL	1 (9)	1 (11)	
Baseline Anti-RBD Ig ≥ 0.8 units/mL	23	22	
Anti-RBD Ig at 8 weeks			0.63
0.8- 100 units/mL	4 (17)	4 (19)	
≥ 100 units/mL	17 (74)	14 (67)	
Missing	2 (9)	4 (18)	

Supplementary Table S6. Alpha diversity metrics.

		Baseline Mean ± SD	Week 4 Mean ± SD	Estimated mean difference (95% CI)	p value
Observed	Inulin	114.11 ± 38.27	99.39 ± 44.92	-13.94 (-41.1 to 13.3)	0.61
	Placebo	118.67 ± 42.48	113.33 ± 38.94		
Shannon	Inulin	3.51 ± 0.57	3.19 ± 0.63	-0.32 (-0.67 to 0.02)	0.13
	Placebo	3.46 ± 0.46	3.51 ± 0.43		
Inverse Simpson	Inulin	21.2 ± 12.64	15.45 ± 10.03	-3.71 (-9.94 to 2.52)	0.46
	Placebo	18.01 ± 10.68	19.16 ± 9.16		
Pielou's Evenness	Inulin	0.75 ± 0.08	0.70 ± 0.09	-0.05 (-0.09 to 0.00)	0.10
	Placebo	0.73 ± 0.05	0.75 ± 0.05		

Supplementary Table S7. Aldex2 differential abundance test between placebo and inulin supplemented KTRs at the genus level.

Genus	Median CLR (centre log-ratio) Placebo	Median CLR (centre log-ratio) Inulin	Effect size	P value of Wilcoxon rank test	Benjamini-Hochberg corrected P value of Wilcoxon test
Bifidobacterium	2.68	5.66	0.511	0	0.02
Anaerostipes	3.32	5.2	0.402	0	0.22
Caproiciproducens	-1.02	1.41	0.411	0.01	0.34
Dialister	-0.945	-0.894	-0.268	0.12	0.73
Prevotella	0.736	-0.752	-0.231	0.09	0.76
Lawsonibacter	-0.945	-0.894	-0.237	0.11	0.78
Fusobacterium	-1.02	-0.959	-0.209	0.18	0.81
Megamonas	-0.945	-0.959	-0.181	0.24	0.82
Raoultibacter	-1.02	-0.784	0.201	0.26	0.83
Enterocloster	3.29	-0.729	-0.23	0.11	0.84
Duodenibacillus	-0.98	-0.959	-0.184	0.3	0.84
Klebsiella	-1.03	-0.959	0.145	0.3	0.85
Mediterranea	-1.05	-0.959	0.116	0.38	0.86
Massilimicrobiota	-1.03	-0.959	0.165	0.33	0.86
Megasphaera	-1.03	-0.99	-0.118	0.35	0.87
Adlercreutzia	-0.98	-0.959	-0.135	0.35	0.89
